# Tri­methyl­phospho­nium *trans*-tetra­chlorido­bis­(tri­methyl­phosphane-κ*P*)iridate(III)

**DOI:** 10.1107/S160053681400350X

**Published:** 2014-02-22

**Authors:** Michael A. Berg, Jesse Davidson, Joseph S. Merola

**Affiliations:** a Department of Chemistry, Virginia Tech, Blacksburg, VA 24061, USA

## Abstract

The title compound, [HP(CH_3_)_3_][IrCl_4_{(H_3_C)_3_P}_2_], consists of a tri­methyl­phospho­nium cation and a tetra­chlorido­bis­(tri­methyl­phosphane)iridate(III) anion. The anion has an octa­hedral arrangement of ligands, with the tri­methyl­phosphane groups occupying *trans* positions. The Ir^III^ atom sits on an inversion center with one P(CH_3_)_3_ ligand and two chloride ligands in the asymmetric unit. The tri­methyl­phospho­nium cation is disordered about a twofold rotation axis. The title compound is the first structurally characterized tetra­chlorido­bis­(phosphane)iridate complex.

## Related literature   

The structure of [((H_3_C)_3_As)ClPd(μ-Cl)_2_IrCl_2_(P(CH_3_)_2_(C_6_H_5_))_2_] can be found in: Briant *et al.* (1981 ▶) (CCDC:530747). The structure of [P(C_6_H_5_)_4_][((H_3_C—CH_2_)_3_P)_2_RhCl_4_] can be found in: Cotton & Kang (1993 ▶) (CCDC:632517). Previous work on ((H_3_C)_3_P)_3_IrCl_3_ can be found in: Merola *et al.* (2013 ▶).
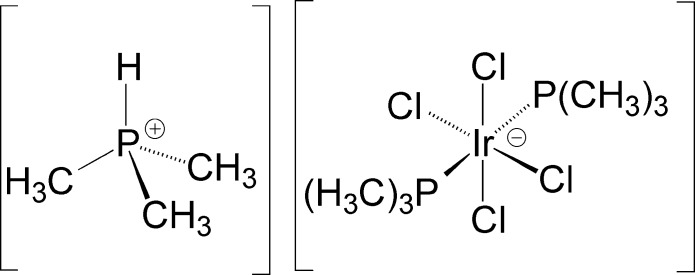


## Experimental   

### 

#### Crystal data  

(C_3_H_10_P)[IrCl_4_(C_3_H_9_P)_2_]
*M* *_r_* = 563.22
Monoclinic, 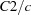

*a* = 15.1814 (5) Å
*b* = 9.8502 (3) Å
*c* = 13.0943 (3) Å
β = 91.843 (2)°
*V* = 1957.09 (9) Å^3^
*Z* = 4
Mo *K*α radiation
μ = 7.60 mm^−1^
*T* = 100 K
0.20 × 0.13 × 0.09 mm

#### Data collection  

Agilent Xcalibur Sapphire3 diffractometer
Absorption correction: Gaussian (*CrysAlis PRO*; Agilent, 2013 ▶) *T*_min_ = 0.360, *T*_max_ = 0.570
10332 measured reflections
3121 independent reflections
2511 reflections with *I* > 2σ(*I*)
*R*_int_ = 0.031

#### Refinement  

*R*[*F* ^2^ > 2σ(*F* ^2^)] = 0.028
*wR*(*F* ^2^) = 0.079
*S* = 0.98
3121 reflections
97 parameters
3 restraints
H-atom parameters constrained
Δρ_max_ = 1.97 e Å^−3^
Δρ_min_ = −0.93 e Å^−3^

### 

Data collection: *CrysAlis PRO* (Agilent, 2013 ▶); cell refinement: *CrysAlis PRO*; data reduction: *CrysAlis PRO*; program(s) used to solve structure: *SHELXS97* (Sheldrick, 2008 ▶); program(s) used to refine structure: *SHELXL97* (Sheldrick, 2008 ▶); molecular graphics: *OLEX2* (Dolomanov *et al.*, 2009 ▶); software used to prepare material for publication: *OLEX2*.

## Supplementary Material

Crystal structure: contains datablock(s) I. DOI: 10.1107/S160053681400350X/zl2577sup1.cif

Structure factors: contains datablock(s) I. DOI: 10.1107/S160053681400350X/zl2577Isup2.hkl

Supporting information file. DOI: 10.1107/S160053681400350X/zl2577Isup3.mol

CCDC reference:

Additional supporting information:  crystallographic information; 3D view; checkCIF report

## Figures and Tables

**Fig. 1.**
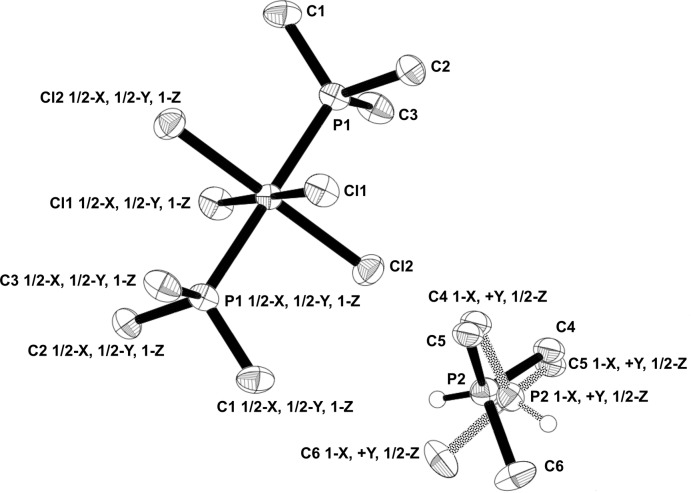
The thermal ellipsoid representation of the fully grown anion/cation pair with atoms labeled with the symmetry operation generating them. Each trimethylphosphonium fragment has 50% occupancy with one fragment indicated by different shading. With the exception of the P—H hydrogen atoms, H atoms are omitted for clarity. The displacement ellipsoids are shown at the 50% probability level.

## supplementary crystallographic information

### 1. Comment

We have been investigating the chemistry of iridium with the strongly electron-donating ligand, trimethylphosphane, for some time. In a recent publication, we discussed how [Ir(COD)(P(CH_3_)_3_)_3_]Cl can be converted into *mer*,tris-(trimethylphosphane)trichloroiridium whose crystals tenaciously hold onto many different solvents (Merola *et al.*, 2013). A direct reaction between IrCl_3_·H_2_O and P(CH_3_)_3_ was attempted to make the same compound in a more direct way, but that reaction did not give a clean product and only a small number of crystals of the title product were obtained.

The title compound crystallizes in the *C*2/*c* space group and the iridium sits on an inversion center. Thus, the iridium (1/2 occupancy), two chlorine atoms and one P(CH_3_)_3_ group are unique with the remainder of the [((CH_3_)_3_P)_2_IrCl_4_] anion being generated by the inversion operator. The cation, trimethylphosphonium, lies slightly offset from a 2-fold rotation axis resulting in a disordered [HP(CH_3_)_3_]^+^ ion where the two sites are generated by the rotation. In aqueous ethanol, reduction of some of the iridium(III)chloride to iridium(I) species will generate HCl and thus lead to the formation of the trimethylphosphonium cation.

This compound is the first crystallographically characterized compound with the [(Me_3_P)_2_IrCl_4_]^-^ ion. The closest analog in the iridium family is a bis-phenyldimethylphosphane complex of iridium with two terminal chlorines and two chlorines bridging between iridium and palladium (Briant *et al.*, 1981). The closest structure to the title iridium compound in the literature is the rhodium analog with triethylphosphane ligands (Cotton & Kang, 1993).

### 2. Experimental

Trimethylphosphane (0.19 g, 2.55 mmol) and IrCl_3_.H_2_O (0.100 g, 0.80 mmol) were refluxed in 95% aqueous ethanol for 3 hr. At the end of that time, the solvent was removed under reduced pressure yielding 0.20 g of a dark, brown, sticky powder. ^1^H NMR spectroscopy indicated that a number of different species were present, possibly with various numbers of PMe_3_ and chloride on iridium as well as a mixture of iridium(III) and iridium(I) species. Attempts to separate different complexes were unsuccessful. A small portion of the solid was dissolved in dichloromethane and the solvent was allowed to evaporate slowly. After evaporation, a very few crystals of the title compound suitable for X-ray diffraction were formed and used for this experiment.

### 3. Refinement

The trimethylphosphonium cation is disordered about a twofold axis and was modeled with each trimethylphosphonium fragment at 50% occupancy. P—C distances within the disordered fragment were restrained to be similar (esd 0.02 Å). Methyl carbon atoms C4 and C5 of the two disordered moieties do overlap substantially and were constrained to have identical ADPs. H atoms were placed at calculated positions and refined using a model in which the hydrogen rides on the atom to which it is attached. For methyl hydrogen atoms *U*_iso_(H) = 1.5*U*_eq_(C). The phosphonium H atom was treated with an idealized tetrahedral geometry (AFIX 13) with *U*_iso_(H) = 1.5*U*_eq_(P).

### Crystal data

**Table:**

(C_3_H_10_P)[IrCl_4_(C_3_H_9_P)_2_]	*F*(000) = 1088
*M**_r_* = 563.22	*D*_x_ = 1.912 Mg m^−^^3^
Monoclinic, *C*2/*c*	Mo *K*α radiation, λ = 0.71073 Å
*a* = 15.1814 (5) Å	Cell parameters from 4635 reflections
*b* = 9.8502 (3) Å	θ = 4.5–31.7°
*c* = 13.0943 (3) Å	µ = 7.60 mm^−^^1^
β = 91.843 (2)°	*T* = 100 K
*V* = 1957.09 (9) Å^3^	Prism, clear light brown
*Z* = 4	0.20 × 0.13 × 0.09 mm

### Data collection

**Table:**

Agilent Xcalibur Sapphire3 diffractometer	3121 independent reflections
Radiation source: Enhance (Mo) X-ray Source	2511 reflections with *I* > 2σ(*I*)
Graphite monochromator	*R*_int_ = 0.031
Detector resolution: 16.0355 pixels mm^-1^	θ_max_ = 32.0°, θ_min_ = 4.1°
ω and π scans	*h* = −22→17
Absorption correction: gaussian (*CrysAlis PRO*; Agilent, 2013)	*k* = −14→14
*T*_min_ = 0.360, *T*_max_ = 0.570	*l* = −18→19
10332 measured reflections	

### Refinement

**Table:**

Refinement on *F*^2^	Primary atom site location: structure-invariant direct methods
Least-squares matrix: full	Secondary atom site location: difference Fourier map
*R*[*F*^2^ > 2σ(*F*^2^)] = 0.028	Hydrogen site location: inferred from neighbouring sites
*wR*(*F*^2^) = 0.079	H-atom parameters constrained
*S* = 0.98	*w* = 1/[σ^2^(*F*_o_^2^) + (0.0438*P*)^2^ + 4.5906*P*] where *P* = (*F*_o_^2^ + 2*F*_c_^2^)/3
3121 reflections	(Δ/σ)_max_ < 0.001
97 parameters	Δρ_max_ = 1.97 e Å^−^^3^
3 restraints	Δρ_min_ = −0.93 e Å^−^^3^

### Special details

**Table:**

Experimental. Absorption correction: CrysAlisPro (Agilent, 2013) Numerical absorption correction based on gaussian integration over a multifaceted crystal model
Geometry. All e.s.d.'s (except the e.s.d. in the dihedral angle between two l.s. planes) are estimated using the full covariance matrix. The cell e.s.d.'s are taken into account individually in the estimation of e.s.d.'s in distances, angles and torsion angles; correlations between e.s.d.'s in cell parameters are only used when they are defined by crystal symmetry. An approximate (isotropic) treatment of cell e.s.d.'s is used for estimating e.s.d.'s involving l.s. planes.

### Fractional atomic coordinates and isotropic or equivalent isotropic displacement parameters (Å^2^)

**Table:**

	*x*	*y*	*z*	*U*_iso_*/*U*_eq_	Occ. (<1)
Ir1	0.2500	0.2500	0.5000	0.02041 (7)	
Cl1	0.10488 (6)	0.33083 (11)	0.52340 (8)	0.0362 (2)	
Cl2	0.26795 (8)	0.22552 (10)	0.67847 (7)	0.0333 (2)	
P1	0.31064 (7)	0.46585 (10)	0.51853 (7)	0.02636 (19)	
C1	0.4083 (3)	0.4741 (4)	0.6014 (3)	0.0372 (9)	
H1A	0.3929	0.4501	0.6713	0.056*	
H1B	0.4323	0.5665	0.6007	0.056*	
H1C	0.4526	0.4104	0.5772	0.056*	
C2	0.2402 (3)	0.5911 (4)	0.5739 (3)	0.0380 (9)	
H2A	0.2182	0.5566	0.6385	0.057*	
H2B	0.1903	0.6101	0.5266	0.057*	
H2C	0.2737	0.6748	0.5866	0.057*	
C3	0.3468 (3)	0.5433 (4)	0.4021 (3)	0.0315 (8)	
H3A	0.3740	0.6314	0.4178	0.047*	
H3B	0.2961	0.5563	0.3549	0.047*	
H3C	0.3900	0.4842	0.3703	0.047*	
P2	0.50757 (18)	0.99525 (19)	0.22786 (14)	0.0272 (4)	0.50
H2	0.5366	0.9618	0.1652	0.033*	0.50
C4	0.5875 (7)	1.0818 (16)	0.3085 (10)	0.0316 (17)	0.50
H4A	0.6290	1.0156	0.3386	0.047*	0.50
H4B	0.5575	1.1293	0.3632	0.047*	0.50
H4C	0.6197	1.1476	0.2678	0.047*	0.50
C5	0.4190 (7)	1.1060 (16)	0.1915 (11)	0.0316 (17)	0.50
H5A	0.3785	1.0591	0.1436	0.047*	0.50
H5B	0.4425	1.1871	0.1587	0.047*	0.50
H5C	0.3873	1.1328	0.2523	0.047*	0.50
C6	0.4725 (6)	0.8538 (9)	0.3010 (7)	0.042 (2)	0.50
H6A	0.5236	0.7979	0.3207	0.063*	0.50
H6B	0.4305	0.7995	0.2599	0.063*	0.50
H6C	0.4440	0.8864	0.3625	0.063*	0.50

### Atomic displacement parameters (Å^2^)

**Table:**

	*U*^11^	*U*^22^	*U*^33^	*U*^12^	*U*^13^	*U*^23^
Ir1	0.02394 (11)	0.02238 (11)	0.01471 (10)	0.00069 (6)	−0.00252 (7)	0.00009 (6)
Cl1	0.0274 (4)	0.0375 (5)	0.0434 (5)	0.0035 (4)	0.0001 (4)	−0.0059 (4)
Cl2	0.0483 (6)	0.0369 (5)	0.0145 (4)	−0.0021 (4)	−0.0047 (4)	0.0010 (3)
P1	0.0321 (5)	0.0243 (4)	0.0223 (4)	−0.0017 (4)	−0.0043 (4)	0.0002 (3)
C1	0.043 (2)	0.031 (2)	0.036 (2)	−0.0080 (17)	−0.0141 (18)	−0.0007 (17)
C2	0.052 (3)	0.0272 (19)	0.035 (2)	0.0029 (18)	−0.0015 (19)	−0.0082 (16)
C3	0.034 (2)	0.031 (2)	0.0292 (18)	−0.0034 (15)	0.0006 (15)	0.0042 (15)
P2	0.0301 (11)	0.0283 (8)	0.0233 (13)	−0.0010 (8)	0.0010 (11)	0.0018 (6)
C4	0.030 (2)	0.021 (5)	0.044 (2)	0.000 (3)	−0.0014 (19)	0.001 (4)
C5	0.030 (2)	0.021 (5)	0.044 (2)	0.000 (3)	−0.0014 (19)	0.001 (4)
C6	0.036 (4)	0.040 (5)	0.050 (5)	−0.014 (4)	−0.014 (4)	0.020 (4)

### Geometric parameters (Å, º)

**Table:**

Ir1—Cl1^i^	2.3717 (9)	C3—H3B	0.9800
Ir1—Cl1	2.3717 (9)	C3—H3C	0.9800
Ir1—Cl2	2.3564 (9)	P2—H2	1.0000
Ir1—Cl2^i^	2.3564 (9)	P2—C4	1.798 (10)
Ir1—P1	2.3264 (10)	P2—C5	1.785 (12)
Ir1—P1^i^	2.3264 (10)	P2—C6	1.781 (7)
P1—C1	1.811 (4)	C4—H4A	0.9800
P1—C2	1.800 (4)	C4—H4B	0.9800
P1—C3	1.807 (4)	C4—H4C	0.9800
C1—H1A	0.9800	C5—H5A	0.9800
C1—H1B	0.9800	C5—H5B	0.9800
C1—H1C	0.9800	C5—H5C	0.9800
C2—H2A	0.9800	C6—H6A	0.9800
C2—H2B	0.9800	C6—H6B	0.9800
C2—H2C	0.9800	C6—H6C	0.9800
C3—H3A	0.9800		
			
Cl1—Ir1—Cl1^i^	180.0	P1—C1—H1C	109.5
Cl2^i^—Ir1—Cl1^i^	89.11 (4)	H1A—C1—H1B	109.5
Cl2—Ir1—Cl1^i^	90.89 (4)	H1A—C1—H1C	109.5
Cl2^i^—Ir1—Cl1	90.89 (4)	H1B—C1—H1C	109.5
Cl2—Ir1—Cl1	89.11 (4)	P1—C2—H2A	109.5
Cl2—Ir1—Cl2^i^	180.0	P1—C2—H2B	109.5
P1—Ir1—Cl1	92.63 (3)	P1—C2—H2C	109.5
P1^i^—Ir1—Cl1	87.37 (3)	H2A—C2—H2B	109.5
P1^i^—Ir1—Cl1^i^	92.63 (3)	H2A—C2—H2C	109.5
P1—Ir1—Cl1^i^	87.37 (3)	H2B—C2—H2C	109.5
P1^i^—Ir1—Cl2^i^	87.56 (3)	P1—C3—H3A	109.5
P1—Ir1—Cl2^i^	92.44 (3)	P1—C3—H3B	109.5
P1—Ir1—Cl2	87.56 (3)	P1—C3—H3C	109.5
P1^i^—Ir1—Cl2	92.44 (3)	H3A—C3—H3B	109.5
P1^i^—Ir1—P1	180.0	H3A—C3—H3C	109.5
C1—P1—Ir1	114.66 (14)	H3B—C3—H3C	109.5
C1—P1—C3	102.8 (2)	C4—P2—H2	109.3
C2—P1—Ir1	115.48 (15)	C5—P2—H2	109.3
C2—P1—C1	102.3 (2)	C5—P2—C4	110.8 (4)
C2—P1—C3	104.5 (2)	C6—P2—H2	109.3
C3—P1—Ir1	115.34 (14)	C6—P2—C4	105.2 (6)
P1—C1—H1A	109.5	C6—P2—C5	112.7 (5)
P1—C1—H1B	109.5		
			
Cl1^i^—Ir1—P1—C1	−45.68 (17)	Cl2—Ir1—P1—C1	45.32 (17)
Cl1—Ir1—P1—C1	134.32 (17)	Cl2^i^—Ir1—P1—C1	−134.68 (17)
Cl1—Ir1—P1—C2	15.72 (17)	Cl2^i^—Ir1—P1—C2	106.72 (17)
Cl1^i^—Ir1—P1—C2	−164.28 (17)	Cl2—Ir1—P1—C2	−73.28 (17)
Cl1—Ir1—P1—C3	−106.51 (15)	Cl2—Ir1—P1—C3	164.50 (15)
Cl1^i^—Ir1—P1—C3	73.49 (15)	Cl2^i^—Ir1—P1—C3	−15.50 (15)

Symmetry code: (i) −*x*+1/2, −*y*+1/2, −*z*+1.
